# Combined use of the hydraulic and hydrological methods to calculate the environmental flow: Wisloka river, Poland: case study

**DOI:** 10.1007/s10661-019-7402-7

**Published:** 2019-03-28

**Authors:** Leszek Książek, Agnieszka Woś, Jacek Florek, Maciej Wyrębek, Dariusz Młyński, Andrzej Wałęga

**Affiliations:** 1 Department of Hydraulics Engineering and Geotechnics, University of Agriculture in Krakow, St. Mickiewicza 24–28, 30–059 Krakow, Poland; 2 Department of Sanitary Engineering and Water Management, University of Agriculture in Krakow, St. Mickiewicza 24–28, 30–059 Krakow, Poland

**Keywords:** Tessman method, Tennant method, Wetted perimeter method, Ichthyofauna habitat requirements, River morphology

## Abstract

The scarcity of water can result in a direct conflict between the protection of aquatic resources and water use. For many agencies, environmental flow (EF) methods are essential in environmental impact assessments and in the protection of important fisheries resources. The objective of this paper is to compare selected hydrological and hydraulic methods and determine the scientifically acceptable and cost-effective way to environmental flow within a section of a mountain river with high naturalness, on the example of the Wisłoka. In this paper, environmental flow was calculated using conventional hydrological methods: Tennant’s, Tessman’s, flow duration curve and hydraulic methods, wetted perimeter method (WPM) and method based directly on ichthyofauna habitat requirements (spawn and migration). The novelty is the combined use of the hydraulic and hydrological methods which relates to flow hydraulics based directly on ichthyofauna habitat conditions. The hydraulic methods provide lower values of environmental flow in comparison with the hydrological methods. The key issue in the use of the hydraulic methods is the choice of criteria. The development of the required set of parameters while taking into account their seasonal nature shifts the method toward habitat modeling methods. However, the scope of habitat requirements of ecosystems must be defined, including the set of aquatic organisms and watercourse type before a hydraulic method may be widely used. Being generally low-cost and simple, the methods presented in this paper can be applied in the water management legislative process.

## Introduction

According to the ecosystem services concept, what man benefits from natural environment is defined as a set of the products and functions of the ecosystem which is used by the community, namely provision, regulation, support, and cultural services (Costanza et al. 1997). When applied to water resources, this relates to the following: (i) fresh waters, generation of electric energy (Operacz 2017), and inland navigation, (ii) flooded areas that reduce the risk of flood and also the cost of its prevention; vegetation (trees, shrubs) prevention of soil losses due to the effect of wind and water (Michalec et al. 2017); marshes that eliminate harmful contaminants, (iii) rivers and estuaries that provide a place for fish reproduction (Strużyński et al. 2015), transfer of water from the soil into plants, (iv) birdwatching, diving and snorkeling, spiritual fulfillment in rivers (Hanson et al. 2008) and in the contact of man with water in a wide sense.

Ecological safety is also connected with the availability of water resources and the threats involved in water deficiency or flood protection. As regards water management, a utilitarian approach is predominant, being deep-rooted in human consciousness; the economic aspect must not be the only criterion of water management (Their 2016).

The key points of water policy by the year 2030, which is intended to prevent water deficiency and drought, comprise the improvement of the retaining capacity of catchments, reconstruction of river continuity and communication with flooded areas, development of flood/drought prevention systems (same actions), water demand management forecasts in drought periods, and water management strategies.

A flow which satisfies water demand both in ecosystems and water-dependent ecosystems is termed “environmental flow” (EF). It is defined as the part of natural flows which should be left in a watercourse and in flooded areas in order to keep high natural values of water ecosystems and water-dependent ecosystems (Tharme 2003). It is calculated as the difference between the observed flow (*Q*) and what is termed instream flow (*Q*_n_). The instream flow is defined as the amount of water which should be kept in the river at its cross-sectional minimum for biological and communal needs (Młyński et al. 2015, as cited in Kostrzewa 1980; Operacz et al. 2018). The parameter relates to the flow in the riverbed only, which is deemed to be its most serious limitation because it ignores the requirements of water-dependent ecosystems such as small water bodies, wetlands, and swamplands. They are an important part of the hydrographic network and affect water circulation processes in a given area (Leibowitz et al. 2008; Lytle and Poff 2004).

More than 200 methods to determine environmental flow have been developed. According to Tharme (2003), methods for the determination of EF can be classified in four (out of six) principal groups: hydrological, hydraulic, habitat simulation, and holistic methods, and two secondary groups: combined and other ones. Other methods are stochastic models based on hybrid spectral and time domain approach for the calibration of shot noise models for daily streamflow generation (Morlando et al. 2016).

Hydrological methods are regarded as the simplest and most easily used ones to calculate EF. They comprise some 30% of all those used. In the hydrological methods, the value of EF depends on the given characteristic flow (Caissie et al. 2014). These methods are based on monthly or daily hydrological records and are recommended as suitable for EF pre-assessment in the water management planning phase. The flow characteristics are relatively easy to determine if time series of daily average flows are available. In their determination, the issues of the naturalization of flows should be considered, i.e., the possibility of not taking into account the use of water, affecting the distortion of the natural hydrological regime of the analyzed watercourse. The environmental flow should specify the requirements for the functioning of ecosystems in relation to the natural hydrological regime. Naturalization of flows is the first problem that applies to hydrological methods that use flow characteristics. The current strong pressure on surface water and groundwater affects disruption of flows (especially low flows). The retention reservoirs, lakes, surface and underground water intakes, sewage discharges (including mine water), as well as morphological transformations, i.e., maintenance and hydrotechnical works will affect the natural hydrological regime of watercourses. In connection with this, the question arises whether the hydrological methods for assessment of environmental flow could get proper flow in regard to habitat requirements. There is also the problem of the accuracy of determining low flows from the stage-discharge curve. Measurements of flow for low stage can be as affected by errors. In the low-state zone, the flow rate curve is often extrapolated due to lack of measurements. This is the second problem with practical use of hydrological methods. How to assess flows in uncontrolled catchments is the third problem. Regional equations are commonly used on the world to asses flow characteristics. On the other hand, the methods give results that can have high error. An example are results presented by Wałęga et al. (2014), where the calculations showed significant differences between the values of LAF (low annual flow) and MAF (mean annual flow) calculated with use empirical formulas and determined on the basis of measurement data. The deviation of the results obtained from empirical equations was between 34 to 67% compare with observed LAF and MAF. A solution in the form of regionalization methods was proposed in Cupak (2017) and Cupak et al. (2017). Therefore, there is a need to introduce methods for calculating the environmental flow, which will not be burdened with errors resulting from flow errors. Of course, in ungauged catchments, conceptual rainfall-runoff models can be used for determinations discharges. Chang et al. (2019) used Neuro-Fuzzy Rainfall-Runoff Models for simulation of daily runoff. The popular model used is the soil moisture accounting (SMA) algorithm (Singh and Jain 2015) or SWAT (soil and water Assessment tool) (Arnold et al. 2015; Chanchal et al. 2017) to continue streamflow simulation. Studies which used the conceptual precipitation-runoff model COSERO, performed by Kling et al. (2015) showed that the model frequently fails when simulations are required outside of calibration conditions in basins with non-stationary conditions. As a consequence, calibration periods should be sufficiently long to include both wet and dry periods, which should yield more robust predictions. It should be remembered that rainfall-runoff models provide only data for calculating the flow characteristics on the basis of which the environmental flow is determined. Hydraulic methods can be such an alternative.

Hydraulic methods (ca. 11% of all) are ones that use quantitative and qualitative relationships between habitats for fish species and flows. The hydraulic methods analyze the effect of increasing flows on the habitats of important fish species in vital phases of their lives, including migration, reproduction, and feeding. Practically, every one out of hundreds of available hydraulic parameters may be used in the methods. The hydraulic parameters which are used for determining EF often include wetted perimeter or maximum depth, usually measured cross-sectionally at such points as are typical of vertical systems. Above all, the hydraulic methods are recommended for use in catchments where hydrometric observations are not carried out, or for controlled cross sections (Efstratiadis et al. 2014)]. The wetted perimeter or maximum depth has been widely used in environmental flow evaluations but there is no conventional, objective method for selecting the critical breakpoint on the curve. As described Gippel and Stewardson (1998), the point is usually chosen solely on a subjective basis, and recommendations can vary between investigators. This was the main reason carried out by the study that was presented in this work.

In Poland, the first attempts to determine the water volume that is required by the aquatic environment were made by Kostrzewa (1977), the author of the formula to calculate instream flow, *Q*_n_. The instream flow is a function of mean annual flow, MLF, and the *k* coefficient which depends on the hydrological catchment type and size: *Q*_n_ = *k* × MLF. In Poland, calculations of environmental flow were carried out for catchment areas ranging from 3.62 to 28,000 km^2^ (Młyński et al. 2015; Piniewski et al. 2010; Wałęga et al. 2015).

In this work, authors used the conventional hydraulic wetted perimeter method (WPM), which indirectly assumes habitat conditions for invertebrates. The novelty is the combined use of the hydraulic and hydrological methods which relates to flow hydraulics based directly on ichthyofauna habitat conditions. It may be a simple way to apply specified ecosystem requirements in detailed scale when decisions of water use have to be made. This method does not require great investments in time and resources. Hughes et al. (2014) emphasized that in situation when neither financial nor the necessary scientific expertise is available then less data-intense and quickly methods may be required. Habitat suitability criteria are the basis of habitat modeling. In this paper, methods with different levels of detail were tested for convergence along a specified river section. We are aware that it is possible to apply more advanced methods of calculating environmental flow, such as holistic or habitat methods (Parasiewicz 2007; Parasiewicz and Adamczyk 2014; Piniewski et al. 2010, 2011). However, as reported by Caissie et al. (2014): “In fact, none of the environmental flow methods have been developed on the basis of tested relationships between the flow regime alteration and ecological responses. As such, many scientists recognize that there are currently no truly scientifically defensible environmental flow assessment methods, as methods are based on common sense rather than scientific proof and validation.” Thus, proposed method is as credible as any other methods, provided that they are applied correctly using the best available information and good judgment. The objective of the research work was to combine use of the selected hydrological and hydraulic methods for determination of environmental flow in a section of a mountain river with high naturalness, on the example of the Wisłok river. Additionally, the novel in the article is the application of a new hydrological approach for calculating environmental flows, proposed by the National Water Management Authority (NWMA) method.

The choice of these two types of methods is not incidental—the cost of their use is the lowest, which is not a negligible criterion in developing new solutions; the only challenge is to make sure the solution is useful for many purposes.

## Materials and methods

### Research area

**Fig. 1:**
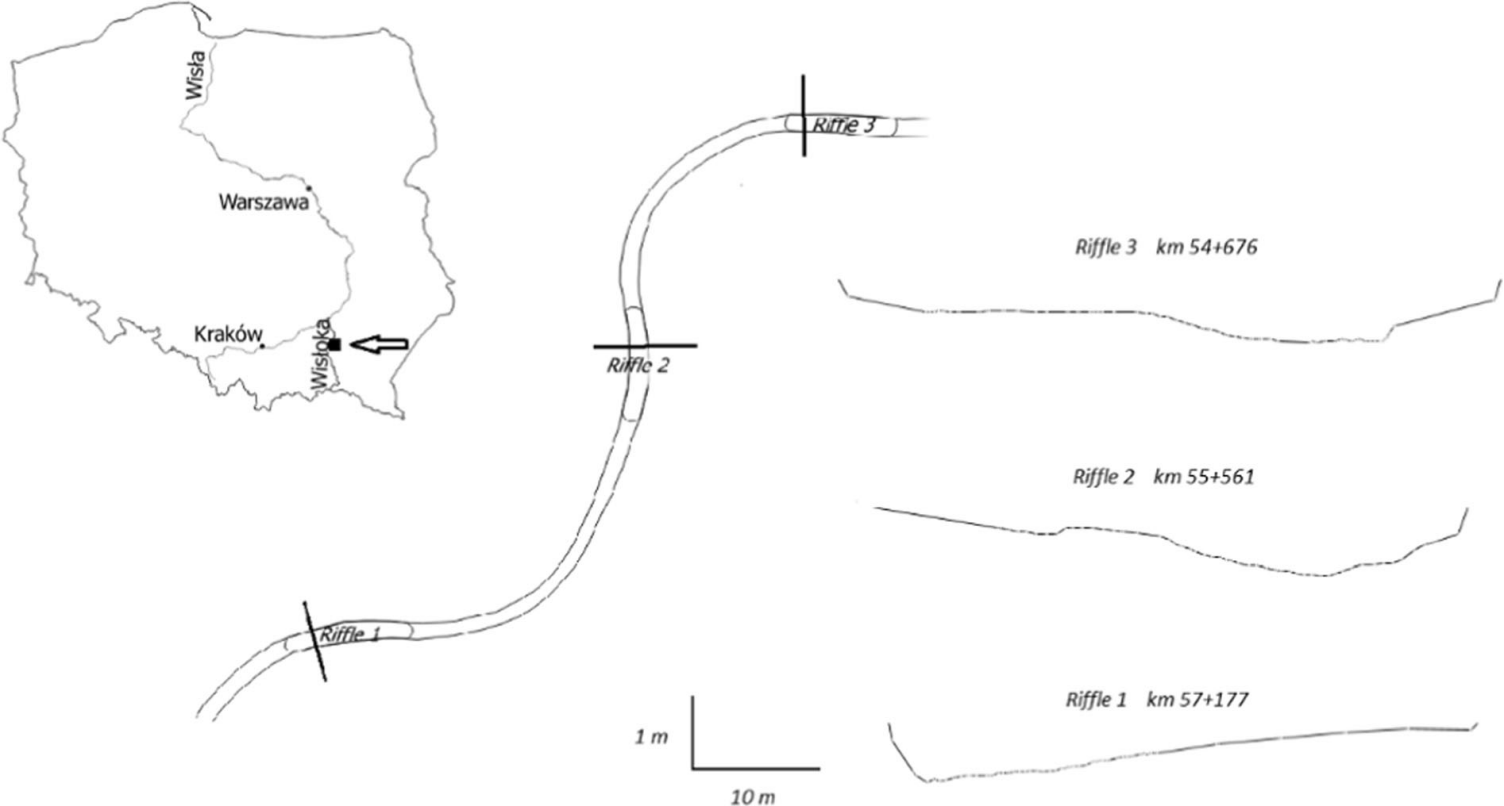
Location of research area

The Wisłoka is a right-hand tributary of the Vistula river, with a length of about 160 km and catchment area of more than 4.1 thousand km^2^. It starts in the Beskid Niski mountain range in the Małopolska province (Fig. 1).

The Wisłoka is one of the rivers where desirable spawning conditions exist for rheophilous diadromous fish species (gravel bottom and water with a suitable oxygen content).

The research section of the Wisłoka is located in the vicinity of the Site of Community Importance, the Lower Wisłoka with tributaries PLH180053. According to the classification of surface water bodies, the research section of the river is located within the natural surface water body RW200019218771 “Wisłoka from the Chotowski Stream to the Rzeka river”, with good chemical and ecological status. The environmental objective of a surface water body is to maintain its good chemical and environmental status and to make the watercourse passable to the aquatic organisms. There is no threat to the attainment of the environmental objectives.

The research section is located below the stage of fall in Dębica (km 57+850). As in a number of Carpathian rivers in the years 1950–1990, the sand-and-gravel bottom of the Wisłoka dropped at an average rate of 5–9 cm year^−1^. The intensity of the process was lower after the year 1990 (Wyżga 2008).

### Hydrological methods

Hydrological methods are some of the most widely used techniques of the assessment of environmental flow. They are based on the assumption that ecological requirements are reflected by hydrological conditions. Determination of environmental flows by hydrological methods is based on hydrological regime indices, such as mean flow MAF, low mean flows MLF, and values resulting from flow duration curves. It is worth noting that, for the respective methods, the values of environmental flows vary significantly (Korsgaard 2006).

The hydrological methods for use in determination of environmental flow comprise two categories: constant flow methods and variable flow methods. The constant flow methods determine a single value of environmental flow, which is valid for the entire hydrological year. That value is found from the flows MAF and MLF. The variable flow methods are used for finding a variable value of environmental flow based on hydrological indices, whereby natural and seasonal flow variabilities are taken into account (Han et al. 2013).

**Fig. 2:**
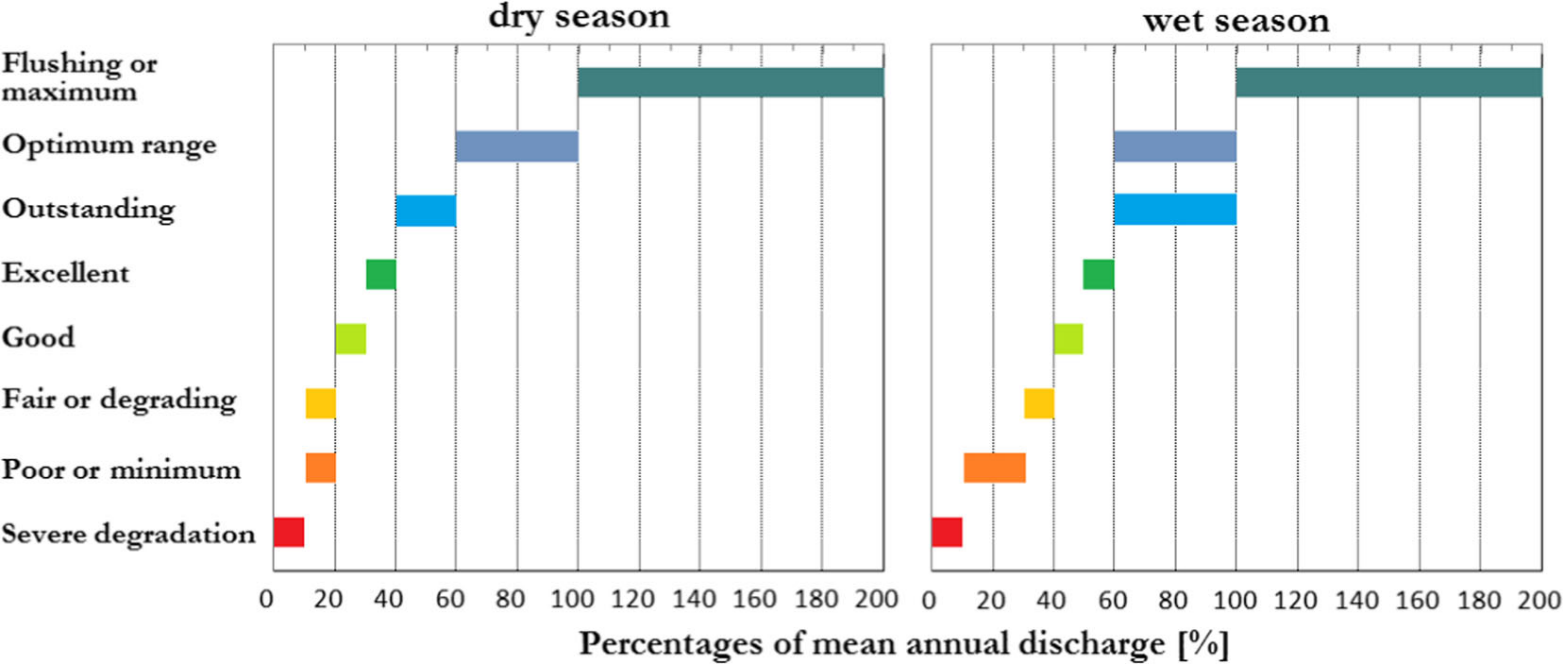
Percentage of mean annual flow required for fish, wildlife, recreation, and related environmental resources in streams flow (Tennant 1976 modified)

For assessment of environmental flow with use of hydrologic methods, the daily mean flows from 1985 to 2015 for Dębica cross section were used. The data were provided by the Institute of Meteorology and Water Management National Research Institute in Warsaw, Poland. In this paper, environmental flow was found by the following hydrological methods: Tennant’s, Tessman’s, and the method based on flow duration curves. Tennant’s method is one of the most widely used and internationally recognized methods of the assessment of environmental flow (Kumar et al. 2010). It enables the establishment of relationships between a given percentage of mean annual flow (MAF) and the quality of ichthyofauna habitats (Fig. 2) (Tennant 1976).

Tessman’s is a modification of Tennant’s method (Tessman 1980). According to the latter, a year is divided in two periods: October to March and April to September, whereas Tessman preferred to divide a hydrological year in 12 monthly periods and classify them in one of three categories, defined by the ratio of mean monthly flow (MMF_mc_) to mean annual flow (Table 1).

**Table 1:** Values of recommended minimum flows according to Tessman

Category	Recommended minimum monthly flow
MMF_mc_ < 0.4MMF	MMF_mc_
MMF_mc_ > 0.4MMF and 0.4MMF_mc_ < 0.4 MMF	0.4MMF
0.4MMF_mc_ > 0.4MMF	0.4MMF_mc_

The method is widely applicable to areas with diversified hydrological and biological cycles (Adhikary et al. 2011). The maintaining of recommended monthly flows is intended to guarantee suitable conditions in the river, enabling the correct course of life processes in the aquatic environment.

The environmental flow was determined from the analysis of flow duration curves for the analyzed river catchments of the Wisłoka river basin. In the literature, the most commonly reported values of environmental flow, identified as low flows, are at the level of *Q*_95%_ and *Q*_90%_ (King et al. 2004). In this paper, the environmental flow was determined as the flow lasting for 90% of the investigated multi-annual period, equal to 10 years.

Environmental flow was also calculated based on hydrological methods proposed by the National Water Management Authority (NWMA 2015). Method involves multiplying the unit low annual flow in bioperiod (*LAF*_*qb*_) by *pb* factor specified for the river type. The basis for determining the *pb* coefficient was a habitat model MesoHabsim (Parasiewicz 2007; Parasiewicz et al. 2013), which on the basis of an analysis of habitat requirements and the historical hydrological data specifies the maximum permissible period of deficit of habitat, and this flow adopts as the minimum environmental flow. The NWMA method is described by formula:

1 $$ EF={p}_b\cdotp {LAF}_{qb}\cdotp A\ \left[{\mathrm{m}}^3\cdotp {\mathrm{s}}^{-1}\right],\mathrm{for}\ \mathrm{gauged}\ \mathrm{catchments}, $$

2 $$ EF=p\cdotp LAF\cdotp A\ \left[{\mathrm{m}}^3\cdotp {\mathrm{s}}^{-1}\right],\mathrm{for}\ \mathrm{ungauged}\ \mathrm{catchments}, $$

where:

*p*_*b*_: parameters of the method for gauged and ungauged catchments (-),
*LAF*_*qb*_: unit low annual flow in bioperiod (m^3^ s^−1^ km^−2^)
*A*: catchment area (km^2^).

For Wisłoka river, four bioperiods were distinguished: I—spring spawning (March–June), II—feeding (July–September), III—autumn spawning (October–December), and IV—winterizing (January–February).

### Statistical verification of data

On the beginning of the analysis, the significance of the trend of the analyzed indices describing short-term freshet flows was assessed by a non-parametric Mann-Kendall test. The Mann-Kendall *S* statistics for a time series is determined from the following equation (Asadieh et al. 2015; Rutkowska and Ptak 2012):

3 $$ S={\sum}_{k=1}^{n-1}{\sum}_{j=k+1}^n\mathit{\operatorname{sgn}}\ \left({x}_j-{x}_k\right) $$

where:

4 $$ \mathit{\operatorname{sgn}}\left({x}_j-{x}_k\right)=\left\{\begin{array}{c}1,\kern0.5em \left({x}_j-{x}_k\right)>0\\ {}0,\kern0.5em \left({x}_j-{x}_k\right)=0\\ {}-1,\kern0.5em \left({x}_j-{x}_k\right)<0\end{array}\right. $$

*n*: number of the time series elements.

Based on normalized *Z* test statistics, as found from the equation:

5 $$ Z=\frac{S-\mathit{\operatorname{sgn}}\ (S)}{Var\ {(S)}^{1/2}} $$

where: Var (*S*)—variance S, defined by the equation:

6 $$ Var(S)=\frac{1}{18}\left(n\bullet \left(n-1\right)\bullet \left(2n+5\right)\right) $$

The probability connected with the normalized Z test statistics was calculated. A trend is regarded as increasing if the Z statistics is positive and is regarded as decreasing if the Z statistics is negative. If the calculated p test probability value is lower than the adopted significance level (*α* = 0.05 in this paper), then the analyzed trend is regarded as statistically significant.

### Hydraulic methods

**Fig. 3:**
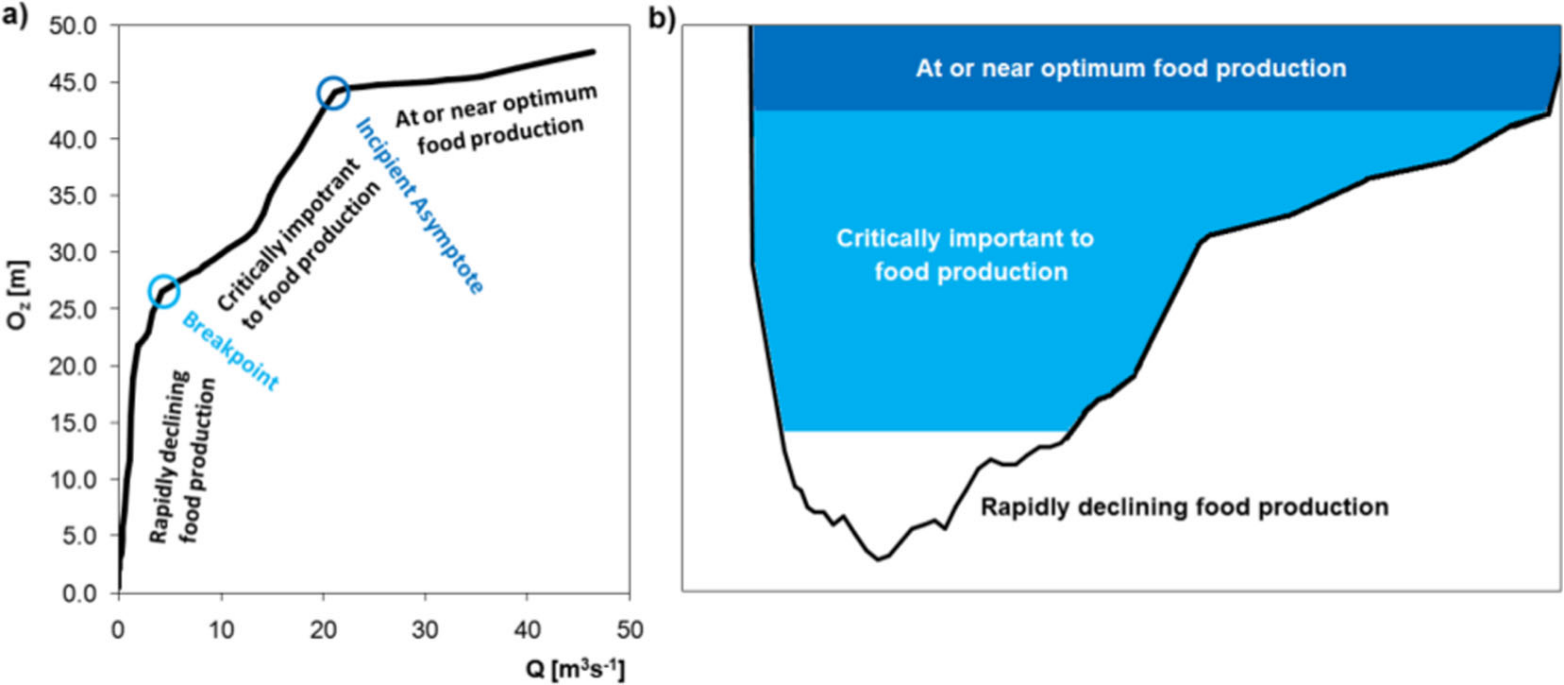
Relationship between hydraulic parameters and invertebrate habitat conditions. **a** Wetted perimeter vs. flow according to WPM. **b** Riverbed filling

The hydraulic methods (or the method of hydraulic indices, also known in the literature as the habitat retention method or the hydraulic geometry method) are office methods, based on relationships between the hydraulic parameters P, e.g., wetted perimeter or depth, and the value of flow in the river. The relationship can be described by the function *P* = a*Q*^b^ where: *Q*—flow; a, b—equation constants, as found empirically for each catchment or as obtained from the graph *P* = *f*(*Q*) (Fig. 3). The reason why the hydraulic method is used for the assessment of environmental flow is the relationship between the hydraulic parameters of watercourses and the quality of the aquatic environment, depending on the requirements of the respective species living in the aquatic ecosystems (mainly ichthyofauna is taken into account in the case of the hydraulic methods) or the direct or indirect connection between the ecological function of the river (Table 2).

**Table 2:** Habitat requirements of flow hydraulics for brown trout

Spawning (Parasiewicz and Adamczyk 2014)		Migration (Bartnik et al. 2011)
Depth (m) d_spaw. min._- d_spaw. max_	Velocity (m s^−1^) v_spaw. min._- v_spaw. max_	Depth (m) d_migr. min_

The wetted perimeter method (WPM) is the most popular of all hydraulic methods. It is based on the relationship between the wetted perimeter for a given cross section of the river and the value of flow with reference to biological requirements. The method is based on the assumption that food availability to fish species is the decisive factor of their existence and condition. Aquatic invertebrates, which breed mainly on riffles, are the essential food for ichthyofauna (Gopal 2013). Riffles as habitats are especially sensitive to changes in flow, responding in changed depths, velocities, and surfaces. The minimum flow which guarantees the appropriate size of a riffle—which is the shallowest habitat in the riverbed—is deemed to be suitable for the entire riverbed including other habitats: pools, runs (Gippel and Stewardson 1998). Riffles are also habitats with the least desirable conditions for the passage of fish species, where the depths are low and the forces generated by the motion of water are considerable. Therefore, the reference cross section for defining the wetted perimeter was selected after the hydromorphological analysis of the riverbed at the highest point of the riffle, where the flow is wide and shallow. The wetted perimeter is defined as the length of the contact path between the cross section and the water (a cross-sectional area perimeter that is “wet” because of the contact with water). Indirectly, this feature defines the habitat surface that is available to the aquatic organisms. Minimum environmental flows are found from the wetted perimeter flow curve, by defining what is called breakpoints. These are the points at which the curvature is the maximum or the slope of the curve changes noticeably; most typically, they are found visually in the graph (Heinz and Woodard 2013). The same effect can be obtained by calculating the curve and tangent slope equation. The breakpoint corresponds to a flow below which the aquatic invertebrate habitat conditions will soon become undesirable as the habitat area decreases. The incipient asymptote defines a flow above which the habitat conditions are optimum (Gippel and Stewardson 1998). Hence, the first inflection point defines critical conditions and the second one does the optimum habitat conditions relative to environmental flow.

The critical riffle analysis, which is a modified WPM, examines the details of migration conditions in riffles. Its result is the suitable depth for a species and the minimum cross-sectional width with the set depth, which is required to enable free passage. In this paper, we have proposed a hydraulic method which is based on detailed ichthyofauna habitat requirements, concerning flow hydraulics and similar to the critical riffle analysis. Based on the assumption of the WPM, that riffles having nearly rectangular cross sections were measured, the mean cross-sectional depth may be analyzed as being representative, without taking into account the width of the belt which provides a suitable depth. Conditions in the spawning season and the minimum conditions for the migration corridor in designing fish ways, as ones which are required also in natural conditions, were considered. Brown trout (*Salmo trutta fario*), which is one of the indicator species in good quality water in gravel-bottomed rivers, was selected as the reference. Habitat conditions define the values of depth and flow velocity which are required in the ichthyofauna spawning and migration seasons. The habitat suitability criteria for brown trout during spawning were applied on the base of MesoHabsim model of Wisłoka river (Parasiewicz and Adamczyk 2014). 0.4 m depth of migration corridor of brown trout was used as minimal value during migration period (Bartnik et al. 2011).

**Fig. 4:**
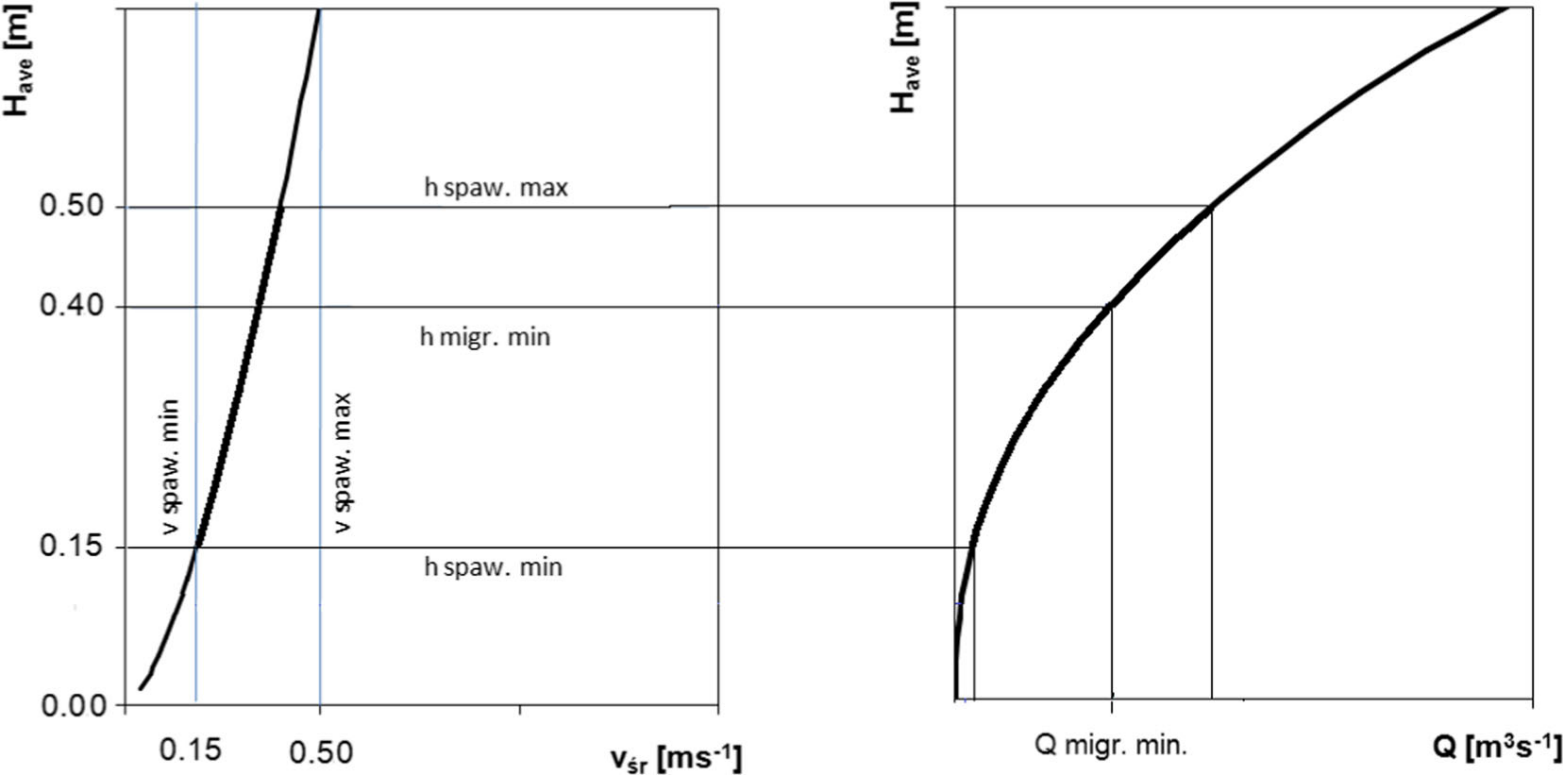
**a** Flow depth—velocity curve with habitat hydraulic requirements for spawning and migration. **b** Depth-discharge curve for determining environmental flow values

Flow values which satisfy these requirements have been obtained by finding the required depths from the depth-velocity relationship and then, for the given depths, determining flow values from the depth-flow curve (Fig. 4). This has resulted in the spawning flow range and the minimum migration flow.

### Field measurements and hydraulic calculations

Environmental flow is connected with specific location. From hydraulic perspective, one cross section may by enough to define flow volume. However, in a situation where more riffles in the section (with no exceed 5% of catchment area) exist, first riffle may not be the most critical. So cross sections in three consecutive riffles within the research section of the Wisłoka river were selected. The cross section is located at the highest points of the riffle. In these locations, fish passage is likely to be limited. Maintenance of flow in these critical cross sections provide river longitudinal connectivity. The measurements included the following parameters: flow (ADCP acoustic Doppler current profiler), water slope, and profile shape. Measurements for the profile shape comprised the exact measurements of bottom configuration and were made using an ADCP probe in the part of the cross section where the depth was > 0.20 m and GPS RTK in the other parts.

The necessary hydraulic relationships were found based on conventional hydraulic calculations. They were then used to find mean velocity and flow for various fillings so as to establish the relationship between the wetted perimeter and flow.

The hydraulic parameters of water flow were calculated using Chezy formula:

7 $$ v=C\bullet {R}_h^{1/2}\bullet {S}_e^{1/2} $$

where: *C*—velocity coefficient $C=\left({R}_h^{1/6}\right)/n$, *R*_h_—hydraulic radius, *R*_h_ *= F/Oz*, *Oz*—wetted perimeter, *F*—cross-sectional area, *n—*Manning’s roughness coefficient obtained from rating curve calibration, *S*_*e*_—energy line gradient (replaced by the water table difference in steady motion conditions). The calculations based on the Chezy formula comprise cross-sectional values of flow and mean depths, whereas the flow hydraulics requirements for habitats relate to local values of flows and depths which are true for the entire habitat area. A riffle is a morphological unit having a uniform bottom with a wide and shallow flow; therefore, it can be assumed that velocities and depths are the same throughout the cross section. This implies that the mean values of hydraulic calculations may be regarded as local for the riffle area.

## Results

### Hydrological calculations

Table 3 shows the results of studies on the flow trend significance, MF, for the Wisłoka river, carried out by means of the Mann-Kendall test. Conclusions were drawn for the significance level *α* = 0.05.

**Table 3:** Results of analysis of flow trend significance, MF for the Wisłoka river

Flow range (m^3^ s^−1^)		Mann-Kendall *S*	Test *Z*	Probability *p*	Trend for the significance level α = 5%
from	to				
18.079	63.474	− 47	− 0.78	0.29	None

Based on the results in Table 3, it has been concluded that no trend exists in the flow observation series MF for the Wisłoka river. This is indicated by the test probability *p*, as found for the Mann-Kendall normalized Z statistics, which is higher than the assumed significance level. Hence, it is concluded that, in the multi-annual period of interest, there has been no factor significantly affecting the hydrological regime of the river.

The results are consistent with those concerning flow trend significance MF for other rivers in the upper Vistula region. In studies reported by Młyński et al. (2015), no statistically significant trends were found in the analyzed characteristic flows. Meresa et al. (2017) indicated a positive trend of different indices derived from hydroclimatic projections for most of Polish and Norwegian catchments. In the light of climate changes having been taking place for some time, the analysis of characteristic flow trends for rivers has become an essential factor in the effective water management planning in a given region (Kundzewicz et al. 2017; Walega et al. 2016).

Basic indices describing the hydrological regime of the river in the multi-annual period 1985–2015 were found, and changes in the dynamics of fluctuations in daily flows in the Wisłoka river have been analyzed. The results of these calculations are shown in Table 4.

**Table 4:** Descriptive statistics for a daily flow observation series for the Wisłoka river, in the multi-annual period 1985-2015

LLF* (m^3^ s^−1^)	MLF* (m^3^ s^−1^)	MAF* (m^3^ s^−1^)	HMF* (m^3^ s^−1^)	C_s_ (−)	Skewness (−)	Kurtosis (−)
1.517	4.601	30.750	1059.836	1.683	7.627	93.386

*LLF the lowest low flow, *MLF* mean low flow, *MAF* mean annual flow, *HMF* the highest maximum flow

Based on the results of these calculations, it was found that daily flow fluctuations in the Wisłoka river were very high in the multi-annual period 1985–2015, as indicated by the value of the coefficient of variation *C*_s_. This is accounted for, first of all, by the alternation of extremely dry and extremely wet years over the entire multi-annual period. The 1980s were a relatively dry decade: the underground water table dropped in the whole territory of Poland, resulting in lower flows in rivers. In contrast, 1997, 1998, and 2010 were extremely wet years with precipitation levels leading to disastrous floods. The obtained value of skewness indicates a right-hand asymmetry of the empirical distribution of daily flows (above zero). Therefore, a majority of hydrometric observations are higher than the value of MAF. On the other hand, the value of kurtosis indicates a leptokurtic empirical distribution of daily flows (positive value of the parameter).

**Fig. 5:**
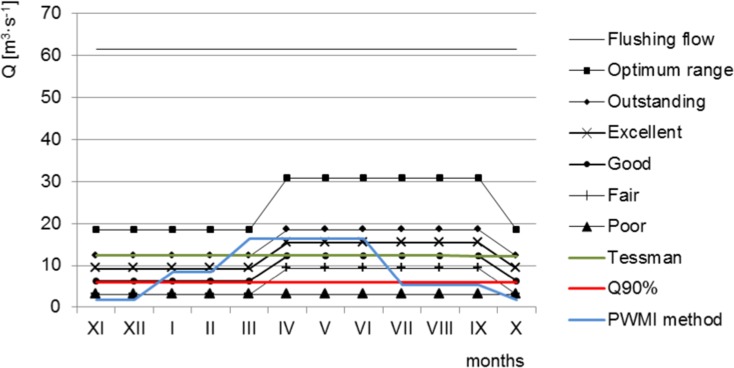
Hydrologically based environmental flow

Because the Mann-Kendall test did not detect any statistically significant trend, in the next step, the hydrometric data (mean daily flows from 1985 to 2015 years) were used for determination of environmental flows for the Wisłoka river. The results of the calculations are shown in Fig. 5. Tennant’s method has enabled determination of the values of environmental flows versus the ichthyofauna habitat requirements. The lowest value in Tennant’s method was 3.075 m^3^ s^−1^ and the highest was 61.5 m^3^ s^−1^ for flushing flow. When using Tessman’s method, environmental flows were found versus mean monthly flows. However, the magnitude between the respective months is not high over the hydrological year: the difference between the minimum and the maximum value is lower than 2%. It is worth noting that the values of environmental flow, as found by Tessman’s method, correspond to the following conditions in Tennant’s method: good for April–September and outstanding for October–March. On the other hand, environmental flow, established as a guaranteed flow at 90%, was 6.025 m^3^ s^−1^ in that multi-annual period; this is comparable to the flow condition good in Tennant’s method. For NWMA method, the lowest value was 1.73 m^3^ s^−1^ (autumn spawning period) and the highest was 16.4 m^3^ s^−1^ (spring spawning period).

**Fig. 6:**
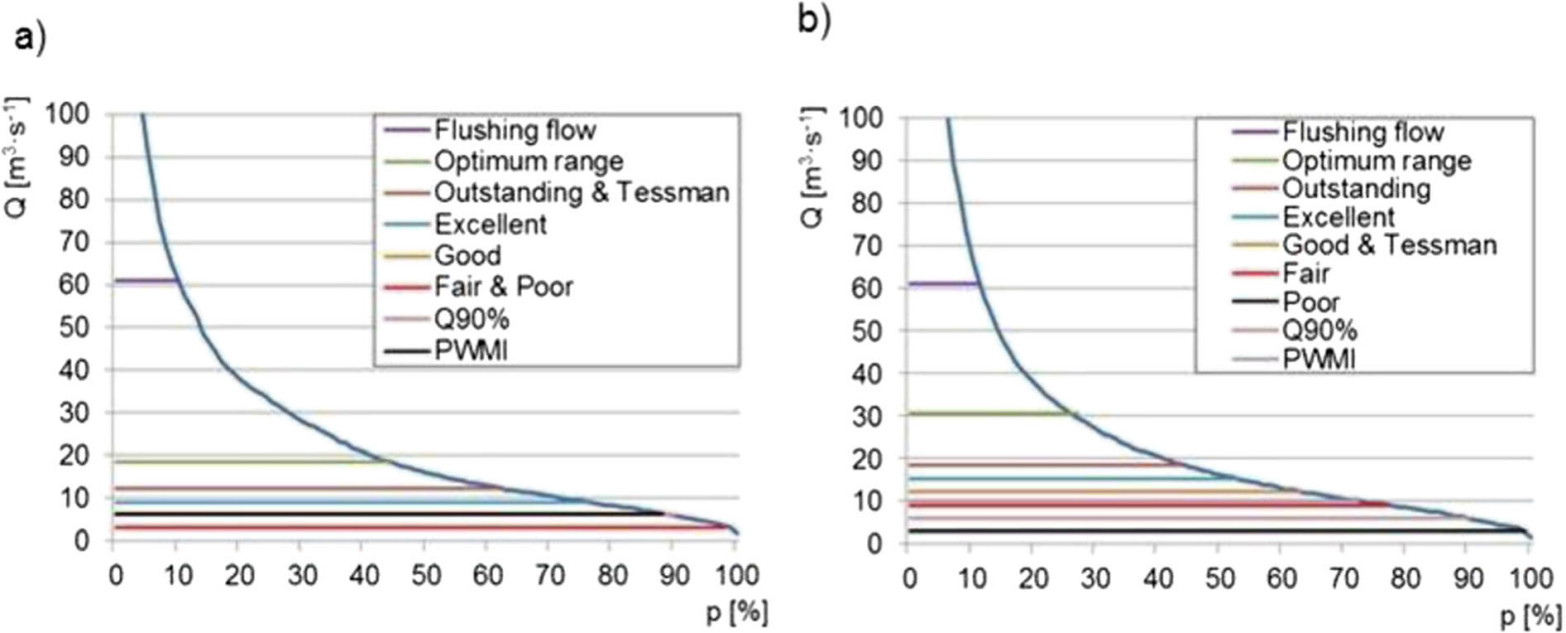
Environmental flows versus flow exceedance time curves for the periods: **a** October–March; **b** April–September

The calculations were followed by an analysis of environmental flows, as found by hydrological methods, versus flow exceedance time curves for the two periods: October to March and April to September. The results are shown in Fig. 6.

An analysis of the possibility of using the Wisłoka river as available water resources indicates that, in the economic aspect, the least desirable solution is to maintain environmental flow at the flushing flow level. It enables water intake for 9.8% time during the year (9.8% 365 day = 36 days per year), on average. The most desirable value in the economic aspect is an environmental flow at the fair and poor level in the winter and poor in the summer. The guaranteed flow for these values is 98%, both in the winter and in the summer. For NWMA (PWMI in fig) method, the guaranteed flow is equal to 89% for autumn spawning and 52% for spring spawning period. From January to July, the flow according to NWMA method was near excellent condition and for the rest of period was—poor to fair conditions—Fig. 5. These results indicate that the value to be adopted is an environmental flow that enables the use of the Wisłoka river water for economic purposes as a potential source of water without degrading the aquatic environment. This is important because the use of water is based on the water management balance, taking into account environmental requirements, among other things. The findings of studies indicate that keeping upper permissible values of environmental flow will lead to deficiencies of these water resources.

### Hydraulic calculations

The results of calculations of environmental flow by means of hydraulic methods are shown in Table 5. The WPM method as well as detailed habitat requirements have shown very low values of critical flow *Q*_1(crit)_ and minimum flow for spawning *Q*_spaw/min_, of less than 1 m^3^ s^−1^. Taking into account the flow width in channel, these values cover between 24 and 31% of the cross-sectional width, relative to the MAF flow width. Surveyed channel provides at least three points of inflection on the curve at each cross section. Higher flow values *Q*_2(opt/crit)_ have been obtained based on the second inflection point previously defined as the beginning of the WMP-based range of optimum habitat conditions: from 3.02 m^3^ s^−1^ for riffle 3 to 3.90 m^3^ s^−1^ for riffle 2; these flows cover a cross section of a width ranging from 43 to 80% of the MAF flow width. The average optimum flow value is about 75% MLF. The third inflection point indicates flow optimum value *Q*_3(opt)_ 11.39 m^3^ s^−1^ and it is in excellent range according to Tennant. The WPM method providing different flow values depends on morphology scale in dispute. The lowest inflection point seems to be disqualified as a breakpoint. In that case, second and third flow values would be defined as critical and optimal respectively, and WPM breakpoint and Tennant’s poor value are similar values (Fig. 5).

**Table 5:** Environmental flow values from hydraulic methods: WPM and Habitat requirements method;.b - percent of MAF flow width

	Wetted Perimeter Method						Habitat requirements method			
	*Q*_1(crit)_		*Q*_2(opt/crit)_		*Q*_3(opt)_		*Q*_spaw_		*Q*_migr_	
	Q (m^3^ s^−1^)	b (%)	Q (m^3^ s^−1^)	b (%)	Q (m^3^ s^−1^)	b (%)	Q_min_-Q_max_ (m^3^ s^−1^)	b_min_ - b_max_ (%)	Q (m^3^ s^−1^)	b (%)
Riffle 1	0.72	29	3.36	43	15.77	96	1.00–25.61	30–99	18.71	97.2
Riffle 2	0.45	31	3.9	74	11.38	97	0.56–12.50	32–97	8.27	89.5
Riffle 3	0.15	24	3.02	80	7.01	97	0.71–17.43	29–98	12.22	96.8
Mean	0.44	28	3.43	66	11.39	97	0.76–18.51	31–98		

**Fig. 7:**
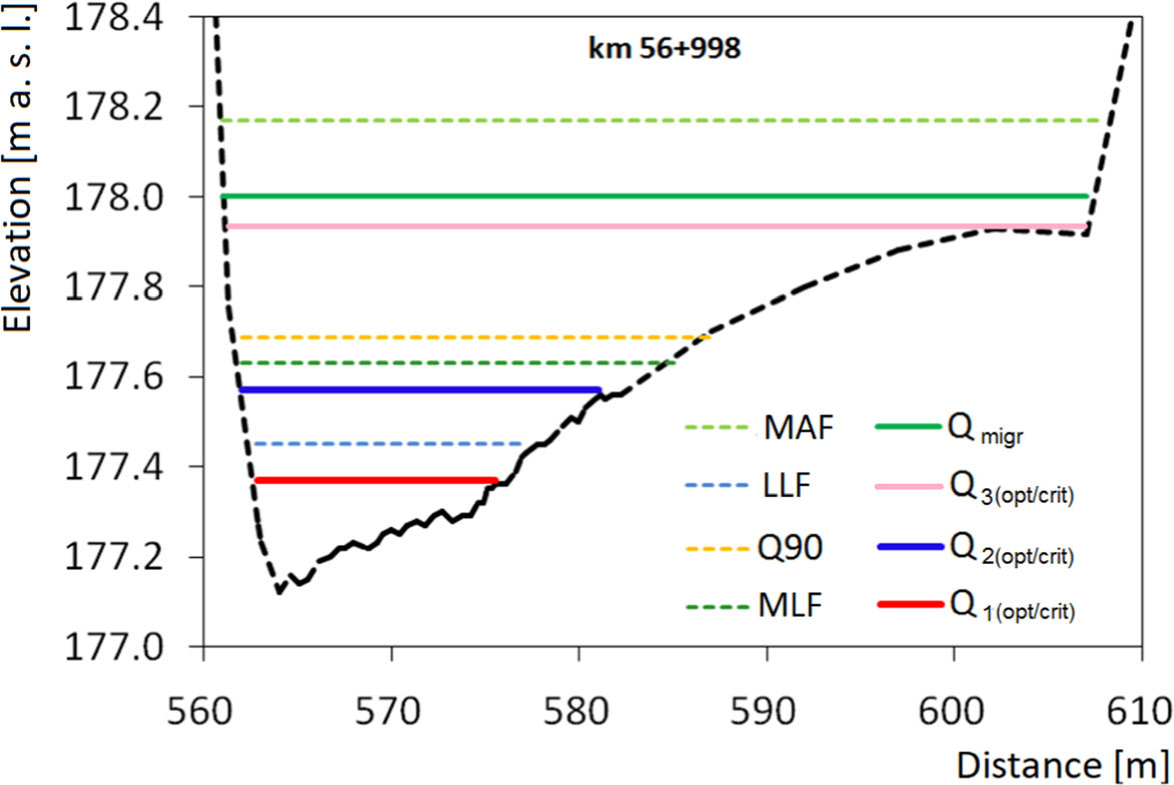
Cross-sectional characteristic flows and environmental flows, riffle 1

In certain phases of their development, aquatic invertebrates and fish species require shallow waters with sufficiently high oxygen contents (Gore et al. 2001); therefore, high flows in the riverbed are not always required. In the aspect of appropriate hydraulic conditions, the minimum flows for trout spawning are lower than LLF about 1 m^3^ s^−1^while the maximum flows range from 17.43 to 25.61 m^3^ s^−1^. According to hydraulic calculations, the flow values which guarantee free passage vary cross-sectionally between 8.27 and 18.71 m^3^ s^−1^; taking into account the nature of passage, the appropriate maximum value must guarantee suitable conditions in all riffles, that is, 18.71 m^3^ s^−1^. At that flow, the riverbed is covered at about 90% relative to MAF. This flow value is higher than offered by third inflection point according to WPM and well in the optimum range according to Tennant. Godinho et al. (2014) suggest that, when calculating values for several locations within a river section, flow is best characterized by stating the mean and the maximum values. We chose to use the mean values except for the migration flow: in that case, the maximum values for three riffles are used. Figure 7 shows the water table levels for the characteristic flows (MLF, MAF, and Q90) and for environmental flows in the watercourse, namely the minimum migration flow and the spawning cross-sectional flow range for the riffle 1.

**Fig. 8:**
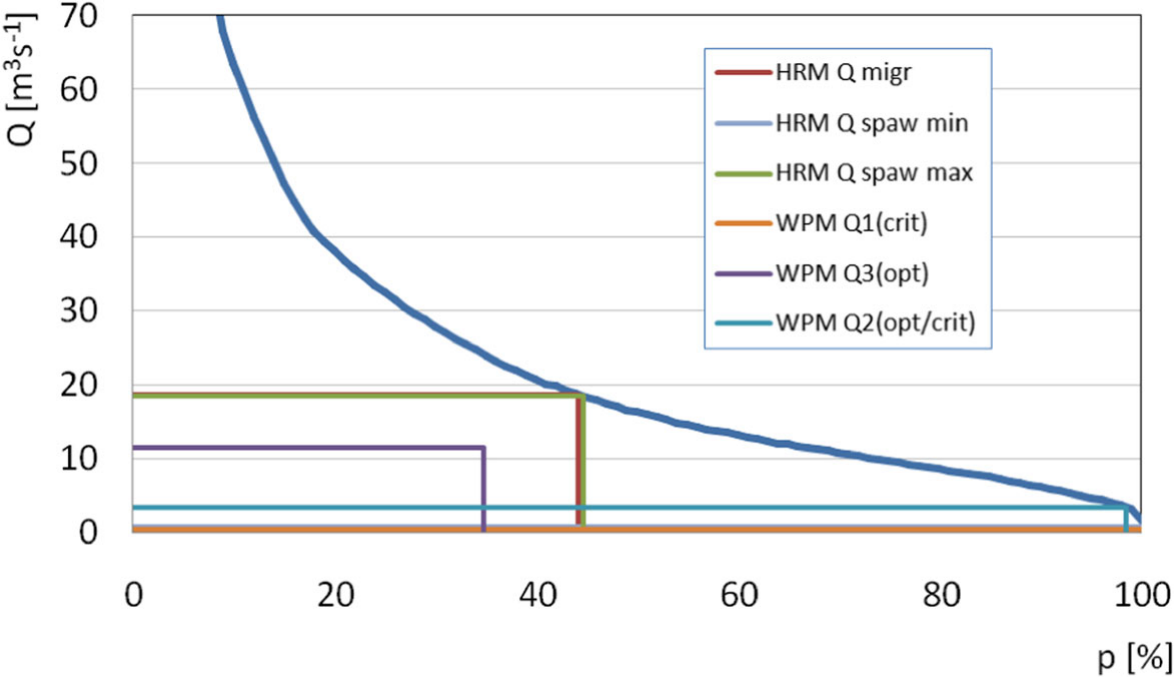
Hydraulically based environmental flows and the multi-annual flow exceedance duration curve

To end with the hydraulic calculations, averaged values of environmental flows provided by the wetted perimeter method and the habitat requirements method were marked on the multi-annual flow exceedance curve for the Wisłoka river (Fig. 8).

Based on the results shown in Fig. 8, the environmental flows resulting from the wetted perimeter method and *Q*_spaw min_ for the habitat requirements method were found to correspond with a 100% time guarantee. Maintaining environmental flows at that level enables the river water intake for economic purposes during the entire year. In the case of *Q*_spaw max_ and *Q*_migr_ for the habitat requirements method, environmental flows corresponded with the occurrence time guarantee of 55 and 80%, respectively. This indicates that water intake is available for an average of 201 and 292 days of the year for the respective values of environmental flows.

**Fig. 9:**
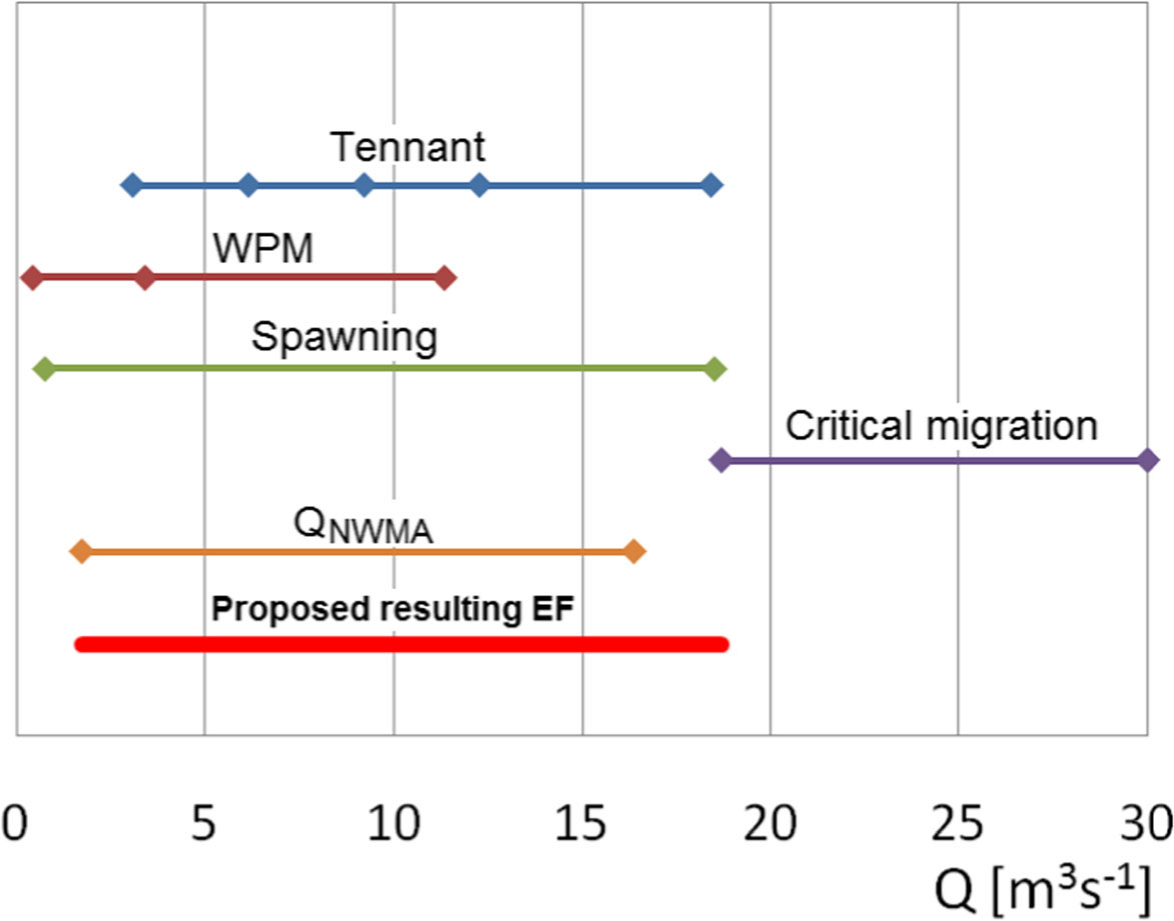
Combination of the results obtained using selected methods

Figure 9 presents combination of selected EF results upon which the final value can be obtained. As we can see, migration taking place at almost any given time in the year cannot be taken into consideration as EF limiting factor. In this way, the remaining methods are used. Also, the lowest breaking point in WPM cannot be used as it is outside the range of other methods. The resulting EF range should be outside the second breakpoint of the WPM method (*Q* = 3.43 m^3^ s^−1^) but inside of the spawning limitations (*Q* = 18.71 m^3^ s^−1^) meeting habitat requirements. In this way, EF will be limited by both hydrological, hydraulic and habitat requirements methods.

## Discussion

The study by Filipek et al. (1987) indicates that the breakpoint in the WPM method occurs at the level of 50% of mean flow. Below that value, the riffles are exposed becoming unproductive, and the river water quality deteriorates. Presented research shows much lower flow values for WPM method about 10% of MAF. Tennant (1976) found that one-half of the wetted perimeter is under water at a mean flow of 10%, and nearly all of the riverbed is filled with water at flows around 30% of mean flow, with the exception of small shallow patches and very wide riffles. Tennant’s observations are confirmed by the present results, which suggest that flows at 10% may cover more than one-half of the wetted perimeter.

In determination of environmental flow, it should be kept in mind that, in certain periods of their lives, aquatic organisms tend to prefer shallow water with sufficiently high oxygen content. Therefore, high flows are not required in their spawning season. The maximum permissible flow value for spawning (18.71 m^3^ s^−1^) is comparable to the minimum flow as found by Tessman’s methods and is higher than 50% MAF. According to Karakoyun et al. (2018), the Tessman method should be preferable rather than the Tennant method in assessment of EF for designing of runoff river–type hydropower plant.

Tennant’s method introduced a division into 2 periods and 8 values ranging broadly between 3.075 and 61.5 m^3^ s^−1^. Tessman’s method, with 12 periods and a scale of 3 values, appears to be less rigorous though cohesive with Tennant’s method. The hydrological method based on the guaranteed flow of 90% is also cohesive with the two methods. It is worth noting that since the use of water is based on the results of the water management balance—which comprises environmental requirements among other things—the approach is an element of the DRIFT method and satisfies its requirements, save for short-term deficiencies, if any.

Among many hydrological methods, the Tennant method and modification is one of the most commonly used in the world for calculating environmental flows (Theodoropoulos et al. 2018). Methods based on MAF flows, assume that in rivers where these flows are similar, the same conditions of the water environment prevail. The *Q*_90_ method represents fair conditions for ecological status. This method is also often used to calculate the environmental flows, as shown by the work of Nilsalab et al. (2017), El-Jabi and Caissie (2019), and Kanclerz et al. 2018. It should be emphasized that hydrological methods to determine environmental flows have some limitations. At the first, the time series of hydrometric data must be available. However, streamflow data are not always available at specific sites of interest (ungauged sites), or the record may be too short to provide particular methods (Ahn and Palmer 2016). This situation can happen because usually the traditional techniques of flow measurements are used, but the traditional approaches may be inadequate to deal with hydrological heterogeneity and complexity. For instance, standard streamflow measurement systems require the deployment of bulky and expensive equipment in the water current along with the assistance of a trained staff. Unfortunately, such requirements have prevented the availability of hydrometric data for many sites. The limitations of traditional monitoring techniques have fostered the use of low-cost equipment such as such as optical cameras (Tauro et al. 2018a; Tauro et al. 2018b). Optical techniques treat flow images as data and apply similarity and pattern recognition algorithms to obtain quantitative information on the flow. Among imaging methods, large-scale particle image velocimetry extracts desired kinematic information by applying high-speed cross-correlation on videos of the surface streamflow. The basic premise of this approach is that a low-cost camera is sufficient to estimate the surface velocity of water streams (Tauro et al. 2016).

Also the NWMA method has some limitations. This method has been developed based on hydrobiological data for 7 rivers (Parasiewicz et al. 2018). Due to the local nature of the correlation between the flow rate and the area of useful (appropriate) habitats for fish, the values of baseflow determined for the given section should not be transferred to cross sections that are not covered by the field studies. There are also significant differences between environmental flows, when the catchment is treated as gauged or ungauged (Pusłowska-Tyszewska and Tyszewski 2018).

In WPM, apparently only one parameter is used on riffles, though, WPM is actually a complex approach which takes into account the riverbed morphology. The WPM flows fit in the Tennant’s range occupying it from significantly lower than poor to the excellent conditions. It is not clear which of the three inflection points is a breakpoint without knowing hydrologic characteristic of the river. Some of research indicates that hydrologic method may provide lower flow values than WPM. Shokoohi and Hong (2011) assume that without considering hydrology, hydraulic and actual situation of the river, application of any of these methods may lead to a wrong conclusion, and cause serious problems for a riverine ecosystem. It should be remembered that hydrological methods are very sensitive to the hydrologic characteristics used in the calculations. The important aspect of both hydraulic methods is to choose proper cross sections—the critical cross sections for longitudinal connectivity to calculation. In a way, results for one section may strongly vary (Lozano et al. 2015; Tare et al. 2017). The example of this approach is the research of Tegos et al. (2017), where representative cross sections for the segment of the river were selected.

Use of rating curve to define water discharge for the required depth may be a concern in aspect of maintaining river longitudinal connectivity from the hydraulic perspective (Yin et al. 2018). In this work, we adopted habitat conditions, provided low minimum requirements for spawning, and at the same time give higher values for migration criteria in the cases of interest. An additional parameter of assessment, that is, the riverbed area under water, combined with the migration criteria improves the method in terms of quality. Its results are cohesive with those provided by hydrological methods. Moreover, owing to its additional criteria—in this case, the use of groups of indicator species and seasonal variabilities—the level of detail is comparable to that provided by habitat modeling methods. Defining flow value from hydraulic model for specified depth requirements was used by Tare et al. (2017) in modifying BBM method. The most important issue of these methods may be to set up the proper habitat criteria values based on ecosystem biology especially in mountain rivers with different environmental variables, e.g., hydrologic alteration (Papadaki et al. 2017).

As regards the spawning and migration criteria, it is worth noting that although they may not be satisfied merely for natural reasons, quite regardless of water consumption, they have different time requirements. Reproduction takes place in a strictly defined period of time, whereas its *Q* range criterion is less restrictive. More restrictive *Q* values with less restrictive time limits are imposed by migration as the provision of contact between concentrations of individuals within a species. We have used several parameters of assessment and several periods of time for a single indicator species, which shows the potential of the hydraulic method and guarantees a number of environmental flow ranges. It should also be taken into account that EF will also be linked with the level of chemical pollution in water (Walling et al. 2017).

## Conclusions

The reasons behind the values of environmental flows may, from the biological point of view, be deterministic (risk of extinction, no option for cost value in the scenario-based holistic approach in the total economy of environmental flow assessment).

In WPM without hydrologic characteristic values of the river, it may be difficult to find out the proper morphology scale to concern.

The habitat requirement method provides width scale of acceptable EF flows based on one species, limited spawning period, and relatively unrestricted timely open migration criteria.

This paper presents the principal groups of methods for determination of environmental flows, including hydrological and hydraulic methods with the aim of finding the EF value in the cost-effective way. The practical result of the study is proposed as an approach where first step is to calculate EF range using Tennant, limit the WPM inflection points to only those in the range of Tennant flows finding between the rest the final EF. In this way, resulting EF would be supported by the morphological conditions of the river.

The essential advantages of the hydraulic method include its low cost, fast and easy use, easy adaptability—this makes it applicable for highly biodiversified river sections. It is also important to take into account the fact that requirements tend to change in the course of the year and to vary with river categories and types. Also in hydraulic methods, a greater number of parameters (discriminants) may be used, and then the scientific discipline of flow hydraulics in open channels becomes a useful tool for their analysis.
